# Fluorine-modified polymers reduce the adsorption of immune-reactive proteins to PEGylated gold nanoparticles

**DOI:** 10.2217/nnm-2023-0357

**Published:** 2024-04-09

**Authors:** Helen Forgham, Jiayuan Zhu, Taoran Zhang, Xumin Huang, Xiangke Li, Ao Shen, Heather Biggs, Gert Talbo, Chun Xu, Thomas P Davis, Ruirui Qiao

**Affiliations:** 1 Australian Institute for Bioengineering & Nanotechnology, The University of Queensland, Brisbane, Queensland, 4072, Australia; 2 Metabolomics Australia (Queensland Node), The University of Queensland, Brisbane, Queensland, 4072, Australia; 3 School of Dentistry, The University of Queensland, Herston, Queensland, 4006, Australia

**Keywords:** fluorination, gold, immune evasion, nanoparticles, polymers, protein corona

## Abstract

**Aim:** To investigate the influence of fluorine in reducing the adsorption of immune-reactive proteins onto PEGylated gold nanoparticles. **Methods:** Reversible addition fragmentation chain transfer polymerization, the Turkevich method and ligand exchange were used to prepare polymer-coated gold nanoparticles. Subsequent *in vitro* physicochemical and biological characterizations and proteomic analysis were performed. **Results:** Fluorine-modified polymers reduced the adsorption of complement and other immune-reactive proteins while potentially improving circulatory times and modulating liver toxicity by reducing apolipoprotein E adsorption. Fluorine actively discouraged phagocytosis while encouraging the adsorption of therapeutic targets, CD209 and signaling molecule calreticulin. **Conclusion:** This study suggests that the addition of fluorine in the surface coating of nanoparticles could lead to improved performance in nanomedicine designed for the intravenous delivery of cargos.

## Plain language summary

Nanomedicines are based around the delivery of therapies by tiny, nanosized delivery vehicles. This method offers a much better way of specifically targeting life-threatening diseases. For fast delivery, nanomedicines can be injected into the blood (intravenously); however, this often leads to an unwanted and exaggerated immune response. The immune system is activated by proteins in the blood that attach themselves to nanoparticles through various chemical interactions (the protein corona effect). Fluorine is a chemical routinely used in surfactants such as firefighting foam and more recently in molecular imaging and nanoparticles designed for the delivery of therapies aimed at cancer. While fluorine has great potential to improve the cellular uptake of therapies, little is known about whether it can also help camouflage the nanoparticles against the immune system responses. Here, using fluorinated polymer-coated gold nanoparticles, the authors demonstrate that fluorine reduces uptake by immune cells and is highly effective at reducing the binding of immune system-initiating proteins. This work successfully illustrates the rationale for more widespread investigation of fluorine during the development of polymer-coated nanoparticles designed for the intravenous delivery of nanomedicines.

## Tweetable abstract

Fluorine-modified polymer coatings on gold nanoparticles exhibit greater antifouling effects against complement and other immunogenic proteins found in the blood.

## Abstract (plain language summary)

The aim of this study was to investigate the influence of fluorine in reducing the adsorption of complement and other immune-reactive proteins onto PEGylated gold nanoparticles (AuNPs).

Reversible addition fragmentation chain transfer polymerization, the Turkevich method and ligand exchange were used to prepare AuNPs capped with polymers containing 0, 3, 6, 15 and 30% fluorine.

*In vitro* physicochemical and biological validations were performed to determine the necessary physiological parameters for each of the AuNPs.

Proteomics was performed to characterize the protein corona fingerprint for each of the five AuNPs.

The adsorption of complement proteins was significantly decreased at 3% fluorine relative to 0% fluorine. The greatest reduction in adsorption was observed at 30% fluorine.

Important immune-reactive proteins (immunoglobulins and platelet basic protein) are also less adsorbed in the presence of fluorine.

Fluorine significantly reduced phagocytosis by macrophages.

CD209 preferentially adsorbs to fluorine-capped AuNPs, suggesting potential future applications for fluorinated polymers as a microbicide or providing a targeted approach for NP delivery of gene therapies aimed at colon cancer.

Calreticulin preferentially adsorbs to fluorine-capped AuNPs, opening up opportunities for NP-led attempts to improve local antitumor immunity.

Collectively, this work successfully illustrates the rationale for more widespread investigation of fluorine during the development of polymer-coated NPs designed for the intravenous delivery of nanomedicines.

Hybrid organic/inorganic nanoparticles have been heavily explored as dual-modality diagnostic agents and delivery vehicles for gene therapies (siRNA, DNA and mRNA) [1–4]. However, current knowledge gaps have, to date, limited the clinical success of these potentially game-changing theranostics. One of the greatest gaps exists within the understanding of nanoparticles and their interaction with proteins located throughout the biological milieu. This process, known as the protein corona effect, is a direct result of the ever-present thermodynamics taking place within the body [5]. It is an inherently complex process of protein adsorption, involving Coulombic and van der Waals forces, hydrogen bonding and hydrophobic interactions and dominated by the uniqueness of any given nanoparticle composition [6].

During corona formation, proteins have been found to modify their normal configuration. Additionally, the most subtle changes in nanoparticle design have been demonstrated to significantly influence how the corona is composed and the potential to create negative effects [7,8]. Such negative effects are routinely evident through poor circulation times and cellular uptake, although most harmful is the off-target cytotoxicity and illicit immune stimulation created by activation of the innate immune system's complement cascade. In reality, this is one of the most common problems faced during intravenous administration of nanomedicines and thus one of the greatest challenges to overcome. Complement proteins deposited onto the surface of nanoparticles primes for clearance by neutrophils and monocytes, but more destructive is the release of anaphylatoxins (C3a, C4a and C5a) capable of promoting a severe adverse reaction in patients, which can be fatal. C5a, in particular, is linked to an acute infusion reaction called complement activation-related pseudoallergy [9,10].

PEGylation, a pathway that involves attaching PEG polymers endowed with various antifouling properties to nanoparticles [11]. This technique has been heavily promoted as a way of preventing protein adsorption and improving overall delivery of nanoparticle platforms [5,12,13]. However, size, charge, shape and surface chemistry nonrelated to PEGylation of nanoparticles may all be responsible for modulating bio–nano interactions and subsequent buildup of a corona [12,14–16]. Additionally, Nandakumar *et al.* show how spontaneous, dynamic changes to the nanoparticle surface over time can be hugely influential in determining the overall corona fingerprint [17].

While corona formation is suggested to be mostly unavoidable, a greater knowledge of how immune reactive proteins interact with the many different chemical building blocks used to engineer PEGylated nanoparticles presents new opportunities to better tune nanoparticles for dual diagnostic and gene-based therapeutic delivery. This knowledge would potentially result in greater control of corona formation *in vivo* by purposefully using chemical building blocks that minimize the buildup of proimmune activating proteins, instead luring proteins to adsorb that improve nanoparticle biodistribution, and priming for increased cellular uptake of nanoparticle-led theranostics [16,18–20]. However, the literature within this area of research remains modest.

Perfluoropolyethers (PFPEs) are building blocks that display chemical and thermal stability properties; they have low toxicity and display low surface energy [21]. Their presence is widely observed in surfactants, where they form hydrophobic surfaces and antifouling coatings [22–24]. Importantly for nanoparticle engineering, PFPEs actively promote the surface repulsion of water during preparation [25,26]. In previous pioneering work, Cheng *et al.* described a multifunctional, tree-like macromolecule modified by a perfluorochemical that enabled consistently high pDNA-delivery efficiency, even after the addition of 50% (v/v) fetal bovine serum (FBS) to the cell culture medium [27]. Also, Yu *et al.* reported a new bioreducible nanofilm with a perfluorochemical core that had good pDNA-delivery efficiency in cells cultured in medium supplemented with 50% v/v FBS. Inclusion of PFPEs can also modulate the surface topology of polymer-coated gold nanoparticles (AuNPs) by increasing hydrophobicity. Surface hydrophobicity of NPs is considered a key factor for their interaction with plasma protein and therefore significantly affects their *in vivo* fate [28]. Most recently, Yang *et al.* reported the synthesis of a glutathione/pH dual activatable crosslinked and fluorinated polyethylenimine with dialdehyde PEG layer loaded with mitochondria-targeting-sequence-KillerRed-superoxide dismutase and clustered regularly interspaced short palindromic repeats/CRISPR/Cas9-carbonic anhydrase IX plasmids for enhanced ferroptosis through endogenous iron dehijacking and *in situ* reactive oxygen species amplification [29]. However, whether the amount of PFPEs present within any given polymer coat is an important factor that helps restrict the adsorption of complement and other immune proteins is not known.

Here, using proteomics to profile the protein corona fingerprint, the authors demonstrate that the percentage of fluorine integrated into the polymer caps of AuNPs significantly reduces complement protein adsorption. They chose AuNPs because they have been widely used as ideal medical and nonmedical material, and they are inert and biocompatible, have low toxicity and can be detected over a broad range of doses with high sensitivity [30–32]. Further, the authors identify that important immune reactive proteins (e.g., immunoglobulins and platelet basic protein) are also less absorbed in the presence of fluorine. Next, they demonstrate how fluorine has the potential to enhance circulatory times and shield against liver toxicity by showing that even 5% of fluorine incorporated within the polymer coat reduces adsorption of apolipoprotein E – previously demonstrated to rapidly remove NPs from circulation and shuttle them straight to the liver [33]. Additionally, they show how anti-inflammatory protein CD209 is more highly adsorbed to fluorinated polymers and discuss how this may be useful as a biologically tuned microbicide or be a beneficial way for naturally targeting gene-based theranostics toward colorectal cancers [34–38]. Finally, they demonstrate an increase adsorption of prophagocytic signaling protein calreticulin and discuss how enhanced adsorption of calreticulin could benefit studies tailored toward increasing tumor-specific immune responses following chemotherapy/radiation [39,40].

## Materials & methods

### Materials

All chemicals and solvents were purchased from Sigma-Aldrich and used as received unless otherwise stated. Hydroxy-terminated perfluoropolyether (PFPE-OH, PFPE AL-2, MW ∼2000 g/mol, Confirmation of Acceptance for Studies number: 126066-30-6) was purchased from the Chemours Company (DA, USA).

### Synthesis of 2-([(butylthio)carbonothioyl]thio)propanoic acid & PFPE-BTPA as the chain transfer agent

2-([(butylthio)carbonothioyl]thio)propanoic acid (BTPA) and PFPE-BTPA reversible addition fragmentation chain transfer (RAFT) agents were synthesized as previously described [41,42]. BTPA (10 mmol), PFPE-OH (5 mmol) and 4-(dimethylamino)pyridine (1.5 mmol) were dissolved in 60 ml of α,α,α-trifluorotoluene and precooled on ice with continued stirring. *N*-(3-[dimethylamino]propyl)-*N′*-ethylcarbodiimide hydrochloride (10 mmol) dissolved in 20 ml of anhydrous dichloromethane was added dropwise to the reaction mixture. The reaction was conducted at room temperature for 48 h. The PFPE-BTPA was obtained by precipitation into a large amount of methanol five times, followed by drying overnight with an oil pump at room temperature. The conversion efficiency of PFPE-BTPA was determined by ^13^C nuclear magnetic resonance (NMR) spectroscopy with a 400 MHz Bruker AC400F spectrometer (dimethyl sulfoxide-d_6_ as solvent).

### Synthesis of block polymers using RAFT polymerization

Block polymers were polymerized using PFPE-BTPA as chain transfer agent (CTA) and monomer oligo(ethylene glycol) methyl ether acrylate (OEGA, Mn 480; Supplementary Table 1). Fluorinated polymers PFPE-Poly(OEGA)_x_ were polymerized using PFPE-BTPA (1.0 equiv.), OEGA (6.0, 16.0, 40.0, 80.0 equiv.) and initiator azobisisobutyronitrile (0.2 equiv.), which were dissolved in 3 ml of α,α,α-trifluorotoluene and 1 ml of *N,N*-dimethylformamide. The solution was then sealed and deoxygenated for 15 min with argon gas. Next, the reaction solution was left in a 70°C oil bath with stirring for 4 h. The final product – PFPE-Poly(OEGA)_x_ – was purified by precipitation in a mixture of diethyl ether and n-hexane (1:1) three times and dried *in vacuo*. The conversion efficiency of PFPE-Poly(OEGA)_x_ was determined by ^1^H NMR spectroscopy (CDCl_3_ as solvent). For nonfluorinated polymer Poly(OEGA)_40_, BTPA (1.0 equiv.) was CTA; OEGA (40.0 equiv.) and initiator azobisisobutyronitrile (0.2 equiv.) were dissolved and synthesized as described above (Supplementary Table 1).

### Preparation of AuNPs

AuNPs were synthesized by the Turkevich method, described previously [43,44]; 100 μl of gold (III) chloride trihydrate (HAuCl_4_ · 3H_2_O; 100 mg/ml) in 100 ml of MilliQ water was heated to boiling and 1 ml of 3% (w/v) C_6_H_5_Na_3_O_7_ was rapidly added to the boiling precursor in the presence of stirring. The reaction was stopped by removing the solution from the heat after it turned bright red.

### Ligand exchange to engraft polymers onto AuNPs

One batch of AuNPs was added to 100 mg of polymer Poly(OEGA)_40_, PFPE-Poly(OEGA)_80_, PFPE-Poly(OEGA)_40_, PFPE-Poly(OEGA)_16_ and PFPE-Poly(OEGA)_6_ separately, synthesizing 0, 3, 6, 15 and 30% fluoro–AuNPs (FNPs). The ligand exchange reaction was performed at room temperature with continuous stirring for 24 h. The final reaction product was centrifuged at 15,000 r.p.m. for 15 min, and the precipitation was resuspended with Milli-Q H_2_O. Three subsequent centrifugation steps were performed to remove any excess free polymers. The concentration of purified product was determined by inductively coupled plasma optical emission spectroscopy (ICP-OES) using the manufacturer's guidelines (iCAP PRO XP, Thermo Fisher Scientific, MA, USA).

### Quantitative analysis of polymer coated onto the AuNPs

The amount of polymer grafted onto the AuNPs was calculated using ^1^H NMR, with 2,2,2-trifluoroethanol (TFE) as internal standard, as previously described [45]. TFE (1 μl) was dissolved in a 500 μl mixture of H_2_O and D_2_O (9:1, v/v) with different concentrations (5, 2.5, 1.25, 0.6, 0.3, 0.06 mg/ml) of polymers. The calibration curves of the polymers were obtained by plotting the integral ratio of polymer at 3.61 p.p.m. and TFE at 3.93 p.p.m. in the ^1^H NMR spectra against the concentrations of the polymers. The AuNPs were then formulated to a final, uniform concentration (0.2 or 0.3 mg/ml) by dilution with 1 μl of TFE in a 500 μl mixture of H_2_O and D_2_O (9:1, v/v).

### Gel permeation chromatography

Gel permeation chromatography analysis of polymer-coated NPs was performed in *N,N*-dimethylacetamide (with 0.03% w/v LiBr and 0.05% 2,6-dibutyl-4-methylphenol) by the Shimadzu modular system comprising a DGU-12A degasser, an SIL-10AD automatic injector and a 5.0 μm bead-size guard column (50 × 7.8 mm) followed by four 300 × 7.8 mm linear Phenogel columns (bead size: 5.0 μm; pore sizes: 105, 104, 103 and 500 Å) and an RID-10A differential refractive-index detector. The temperature of the columns was maintained at 50°C using a CTO-10A oven, and the flow rate was kept at 1 ml/min using an LC-10AT pump. A molecular weight calibration curve was produced using commercial narrow molecular weight distribution polystyrene standards with molecular weights ranging from 500 to 106 g/mol. Polymer solutions at 2–3 mg/ml were prepared in the eluent and filtered through 0.45 μm filters prior to injection.

### Interfacial tension of polymer-coated AuNPs

The hydrophobicity of polymer-coated AuNPs with different fluorine contents was tested by time-dependent tensiometer out at the water–air interface. The dynamic surface tension of the FNPs at the water–air interface was measured using the pendant drop method (OCA20, Dataphysics, Stuttgart, Germany). All experiments were performed at room temperature (23 ± 1°C). The equilibrium interfacial tension (IFT) was recorded by time-dependent dynamic surface tension plots, where the equilibrium surface tension approached the equilibrium value after time. The IFT test monitors up to 5 min and measures every 2 s.

### UV-VIS chromatography

All UV-VIS spectra were acquired by a Shimadzu UV-2600 UV-VIS-near infrared spectrophotometer in quartz cuvettes of 10 mm path length, as previously described [46].

### Transmission electron microscopy

Transmission electron microscopy (TEM) was used to observe the morphology of the NPs. TEM images were obtained by a Hitachi HT7700 equipped with a tungsten filament, as previously described [47].

### Dynamic light scattering

The hydrodynamic size of the AuNPs in different solvents (MilliQ H_2_O, phosphate-buffered saline [PBS] and DMEM) was analyzed by a Nano ZS scanner (Malvern, UK) at 298.0 K. DTS-0012 cuvettes were used for taking measurements at a wavelength of 632.8 nm. The polydispersity index describing the width of the particle size distribution was measured and found to be below 0.3, the expected polydispersity index for polymer NPs. Measurements were carried out for each sample in triplicate and are presented in graphed format using the intensity-weighted size distribution method [48].

### Cell culture

Chinese hamster ovary CHO-A5 cells (Life Technologies, Gibco-BRL, MA, USA) were used as received. DMEM supplemented with 10% FBS, 1% pen–strep, 1% L-glutamine, 1% nonessential amino acids, 50 mg/ml Geneticin and modified Eagle medium supplemented with 10% FBS were used to culture CHO-A5. RAW 264.7 (TIB-71) and MCF-7 (HTB22) cells were purchased from American Type Culture Collection (VA, USA) and used as received. Cells were grown in DMEM supplemented with 10% FBS and 1% pen–strep. All cells were incubated in a humidified atmosphere of 5% CO_2_ at 37°C and passaged at a confluency of 70%.

### Cytotoxicity

The potential cytotoxicity of the AuNPs was determined by alamarBlue assay. Briefly, CHO-A5 and MCF-7 cells were seeded in 96-well plate at a density of 10,000 cells per well. After 24 h, the cells were administered increasing concentrations of NPs based on Au content (0, 0.005, 0.01, 0.02, 0.05, 0.1 μg/ml) and incubated for 24 h. The following day, alamarBlue reagent (10%) was added to the cells, followed by incubation at 37°C with 5% CO_2_ for 4 h. Fluorescence intensity at 560/590 nm was measured using the EnSight plate reader (PerkinElmer, MA, USA).

### Uptake studies in macrophages

To determine whether fluorinated polymers influence phagocytic uptake by macrophages, the 0, 3, 6, 15 and 30% fluorine polymer-capped NPs were incubated (100 μg/ml) with mouse macrophage cell line RAW 264.7 for 24 h. Following incubation, the cells were pelleted and sent for ICP analysis performed on the Optima 8300 (PerkinElmer). Uptake was also examined by dark field imaging using the Olympus DSX-1000 digital microscope set to a 50× objective lens (Olympus, Japan).

### Collection of plasma proteins

Human blood samples were procured in accordance with the approval granted by the ethics committee under reference number 2021/HE000391. Blood specimens were obtained from three donors and sourced from the Australian Red Cross. All experiments were performed according to relevant laws and institutional guidelines of the University of Queensland Health & Safety Committee.

Briefly, the blood was divided into 50 ml tubes and subjected to centrifugation at 4°C, 2000× g for 15 min. During this process, the cellular components of the blood were separated and removed, leaving only the plasma component. The isolated plasma was carefully portioned into 15 ml tubes and promptly stored at -80°C.

### Protein corona collection

Samples for corona analysis were prepared using previously published methods [49]. Briefly, all AuNPs were diluted to 1 mg/ml in PBS, then diluted with plasma to make 1 ml of 0.2 mg/ml AuNPs. The AuNP/plasma samples were incubated on a shaker at 37°C for 24 h. The samples were then centrifuged at 16,300× g for 15 min and the protein corona pellets collected. Each pellet was washed with 10 mM PBS thrice, followed by one-time washing using NaHCO_3_ to remove any remaining supernatant before being sent to Queensland Metabolomics and Proteomics for proteomic analysis.

### Proteomic analysis

Samples were redissolved in 20 μl loading buffer 5% acetonitrile, and 2 μl was injected into a trap column (Thermo Fisher Scientific Aquasil 5 μm C18 HPLC column) at a flow rate of 10 μl/min. Following a 3 min wash step, the column was switched in line with a resolving column (Water nanoEase 100 mm × 150 μm, 1.8 μm, 100Å). Using a gradient, samples were eluted at 8% over 4 min. This was followed by 24% at 47 min, 40% at 53 min and finally 95% at 57 min. Mass spectrometry (MS) using liquid chromatography–MS/MS was performed using a Thermo Fisher Scientific ultra-HPLC system coupled to an Exploris 480 mass spectrometer with an FAIMS Pro interface. The FAIMS compensation voltages were -45 V and -65 V. The electrospray voltage was 2.2 kV in positive-ion mode, and the ion transfer tube temperature was 295°C. Full MS scans were acquired in the Orbitrap mass analyzer over the range of m/z 340–1110 with a mass resolution of 120,000. The automatic gain control target value was set at ‘Standard’, and the maximum accumulation time was ‘Auto’ for the MS. The MS/MS ions were measured in six windows from mass 350–470, in 18 windows from mass 465–645 and in five windows from mass 640–1100 with an overlap of 1 m/z and quadrupole isolation mode. Analyses of the data were performed using Spectronaut against a reference proteome with a Q-value cutoff of 0.05.

## Results

### Chemical design & synthesis of macromolecular surface ligands

**Figure 1.**
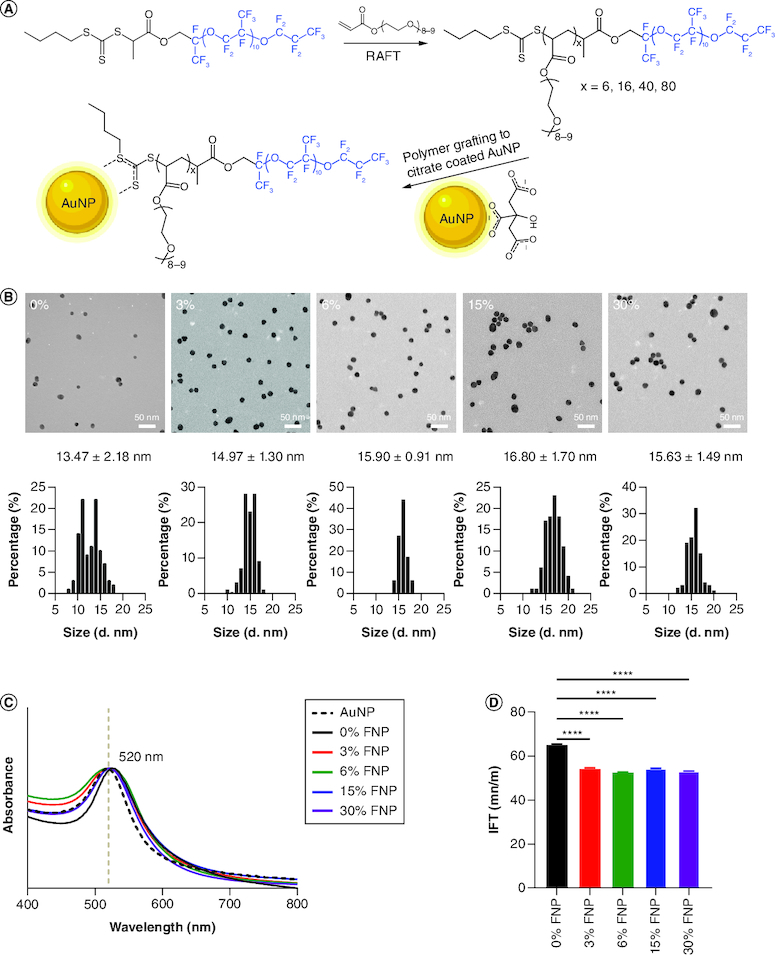
Characterization of polymer-coated gold nanoparticles. **(A)** Synthesis of polymers and surface grafting of gold nanoparticles by fluorine polymers. **(B)** Transmission electron microscopy and histography of polymer-coated gold nanoparticles. **(C)** UV-VIS of polymer-coated gold nanoparticles from 400 to 800 nm. **(D)** Interfacial tension of polymer-coated gold nanoparticles at the water–air interface. ****p ≤ 0.0001; one-way analysis of variance, Dunnett's multiple comparisons test. AuNP: Gold nanoparticle; FNP: Fluoro–gold nanoparticle; IFT: Interfacial tension; RAFT: Reversible addition fragmentation chain transfer.

The chemical design of the AuNP macromolecular ligands was based on a brush polymeric structure. It combined a trithiocarbonate group for anchoring to citrate anions at the particles' surface and a fluorine brush polymer composed of repeating units of OEGA [50]. As shown in Figure 1A, the polymers were synthesized through RAFT polymerization using a fluorinated (PFPE-BTPA) or nonfluorinated (BTPA) CTA. PFPE-CTA was reacted with OEGA monomer at different degrees of polymerization depending on the percentage of fluorination required. Subsequent RAFT, radical polymerization resulted in the development of four individual fluorinated block polymers with a fluorine content approximately 3, 6, 15 and 30%, respectively. The 0% fluorine polymer was composed of monomer OEGA and BTPA-CTA (degrees of polymerization = 40). The polymers were thoroughly characterized by ^1^H NMR (Supplementary Figures 1–5) and size exclusion chromatography (Supplementary Figure 6). NMR maps for each polymer demonstrate successful polymerization illustrated as characteristic peaks, with the ^1^H x-peak accurately detecting the OEGA content within each of the five polymers (Supplementary Figures 1–5). The polydispersity (*Đ*) and molecular weight of synthesized polymers were characterized by size exclusion chromatography and are summarized in Table 1 & Supplementary Figure 6. All polymers displayed similar retention times (Supplementary Figure 6). The molecular information of the polymers is summarized in Table 1; in each instance, the percentage conversion rate was greater than 94% with a narrow molecular weight distribution (*Đ* <1.21; Table 1).

**Table 1.** Experimental conditions and molecular weights of polymer, determined by gel permeation chromatography.

Polymers	Degrees of polymerization	Conversion	Fluorine content	*M*_n:GPC_ (kDa)*^a^*	*M*_w:GPC_ (kDa)*^a^*	*Đ^a^*
Poly(OEGA)_40_	40	99%	0%	14.3	17.3	1.21
PFPE-Poly(OEGA)_80_	80	99%	3%	28.9	34.7	1.20
PFPE-Poly(OEGA)_40_	40	97%	6%	12.0	14.2	1.18
PFPE-Poly(OEGA)_16_	16	98%	15%	7.6	8.4	1.11
PFPE-Poly(OEGA)_6_	6	94%	30%	5.0	5.3	1.06

GPC: Gel permeation chromatography; OEGA: Oligo(ethylene glycol) methyl ether acrylate; PFPE: Perfluoropolyether.

### Characterization of polymer-coated AuNPs

**Figure 2.**
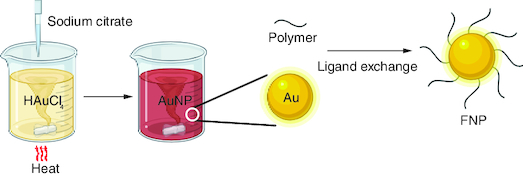
Chemical synthesis of gold nanoparticles using the Turkevich method. AuNP: Gold nanoparticle; FNP: Fluoro–gold nanoparticle.

The Turkevich method was used to prepare colloidally stable AuNPs (14.47 ± 1.21 nm) by reduction of the gold salt with sodium citrate in water (Figure 2) [51]. Polymer-coated AuNPs were prepared using a ‘grafting to’ approach featured by citrate reduction of the AuNP citrate surface to the trithiocarbonate group of polymer [50]. 100 mg of polymer was added to the AuNP suspension and reacted over 24 h to produce 0, 3, 6, 15 and 30% fluorine polymer-capped AuNPs suspended in H_2_O. Successful polymer engraftment was calculated using NMR – referencing AuNPs as a control (Supplementary Table 2). Using test concentrations of 0.2 mg/ml for 0, 3 and 6% and 0.3 mg/ml for 15% and 30% FNPs, any differences in polymer concentration for each NP formula were negligible (0.17, 0.15, 0.10, 0.13 and 0.20 mg/ml, respectively). However, the fluorine content (%) of the particles was predictably higher in accordance with the percentage of fluorine in the fluorine polymer coating, with a tenfold increase observed between the 3% and 30% (2.1% and 20.0% content, respectively), which revealed a similar surface coverage of particles. As displayed in Figure 1B, FNPs were characterized by TEM, which were identified as uniform, spherical NPs with no observable aggregation. The average size of each NP starting from 0%, subsequently increasing in fluorine content within the polymer coat was 13.47 ± 2.18, 14.97 ± 1.30, 15.90 ± 0.91, 16.80 ± 1.70 and 15.63 ± 1.49 nm, respectively (n = 100 NPs per sample measured), which did not display a size or shape change compared with AuNP particles (14.47 ± 1.21; Supplementary Figure 7). UV-VIS spectroscopy produced sharp peaks indicative of a monodispersed population, with a consistent ∼520 nm peak in absorbance representing AuNPs of ∼20 nm size in all samples (Figure 1C). The graphed UV-VIS data show a slight, expected, nanometer shift to the right for the polymer-capped AuNPs relative to the AuNP, indicative of effective polymer engraftment.

The authors next examined water surface tension as a measure of hydrophobicity [22]. Here, they implemented a time-dependent pendant drop tensiometer to measure the IFT at the water–air interface (0% fluorine polymer-capped AuNPs as the control; Figure 1D). The IFT was recorded through the time-dependent dynamic surface tension plots, where the IFT approached an equilibrium value after a certain time (Supplementary Figure 8). As shown in Figure 1D, in the absence of fluorine, the IFT of the control was significantly higher (65.27 mN/m; p < 0.0001), indicating decreased hydrophobicity at the particle surface [52]. While fluorination reduced the IFT, no correlation with increased fluorine percentage was recorded (54.36, 52.41, 53.89, 52.87 mN/m, respectively; Supplementary Table 3).

### Size & colloidal characterizations in biologically relevant solutions

**Figure 3.**
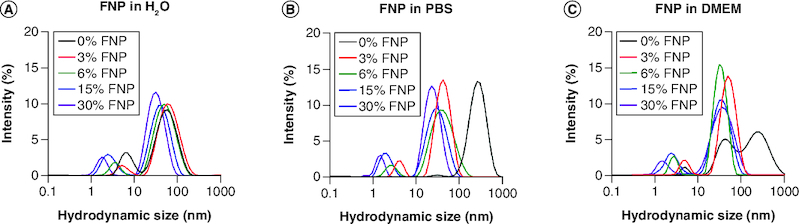
Colloidal stability of fluoro–gold nanoparticles. **(A–C)** Hydrodynamic size distribution of polymer-coated gold nanoparticles in H_2_O, phospahte-buffered saline and DMEM, respectively. DMEM: Dulbecco's modified eagle medium; FNP: Fluoro–gold nanoparticle; PBS: Phosphate-buffered saline.

The colloidal stability of FNPs was examined using dynamic light scattering, whereby the NPs were suspended in PBS, DMEM or H_2_O as a solution control. In H_2_O, uncoated particles showed a diffuse pattern, rather than distinctive peaks of distribution, with an average size of 126 nm when using the size distribution by intensity percentage method of analysis (Supplementary Figure 9A). All polymer-capped AuNPs displayed two distinct, individual distribution peaks (Figure 3A). The largest peaks most representative of the main concentration of NPs within the solution are shown in Table 2 and are based on the area under the curve analysis in GraphPad. Fluorine in the polymer caps causes adaptation to the NP surface, as well as potential alterations to the routine properties of polymer-capped AuNPs [53]. Collectively, this led to a decrease in the overall hydrodynamic size. Most notable was the change observed between the 3% and 30% FNPs, which resulted in a hydrodynamic size reduction from 59 nm to 32 nm. When the bare AuNPs were submerged in PBS, the distribution pattern changed from diffuse to a singular, observable peak within the GraphPad data (Supplementary Figure 9B). Additionally, the average hydrodynamic size within the population was increased from 126 nm to 170 nm. This was also true for the non-FNPs, which changed in hydrodynamic size from 59 nm to 268 nm. However, for the FNPs, the opposite was observed. Each NP had the same two characteristic peaks; however, rather than increasing in size, all five NPs reduced in size, compared with when they were in H_2_O (44 nm, 38 nm, 32 nm and 24 nm, respectively; Table 2 & Figure 3B). When the NPs were added to DMEM (no FBS), the AuNPs' hydrodynamic size increased further to 198 nm, whereas the non-FNPs stabilized at 267 nm. However, the FNPs' size was more variable, and for the 15% and 30% FNPs, an increase in size was observed (Table 2, Supplementary Figure 9 C & Figure 3C).

**Table 2.** Hydrodynamic size of polymer-capped nanoparticles in physiologically relevant solutions.

Fluorine%	Hydrodynamic size in H_2_O	Hydrodynamic size in phosphate-buffered saline	Hydrodynamic size in DMEM
0%	59 nm	268 nm	267 nm
3%	59 nm	44 nm	51 nm
6%	51 nm	38 nm	32 nm
15%	43 nm	32 nm	38 nm
30%	32 nm	24 nm	38 nm

DMEM: Dulbecco's modified eagle medium.

### Cytotoxicity of FNPs

**Figure 4.**
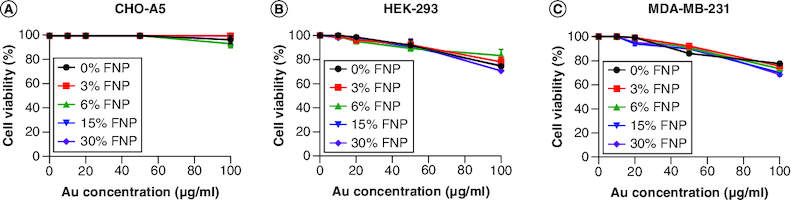
Cytotoxicity of polymer-coated gold nanoparticles. Cytotoxicity of polymer-coated gold nanoparticles on **(A)** CHO-A5, **(B)** HEK-293 and **(C)** MDA-MB-231 cell lines after 24 h incubation, n = 3 independent experiments. FNP: Fluoro–gold nanoparticle.

To confirm that the 0, 3, 6, 15 and 30% fluorine polymer-capped AuNPs were nontoxic to cells, the authors examined the health of αIIbβ3-expressing Chinese hamster ovary (CHO-A5), human embryonic kidney (HEK-293) and human breast cancer MDA-MB-231 cell lines by alamarBlue assay 24 h after incubation at escalating concentrations (0, 5, 10, 20, 50, 100 μg/ml). The FNPs showed a negligible effect on the viability of CHO-A5 cells up to a gold concentration of 100 μg/ml. Slight toxicity was observed on HEK-293 and MDA-MB-231 cells at 100 μg/ml, albeit less than 25% overall (Figure 4A–C).

### Phagocytic uptake

**Figure 5.**
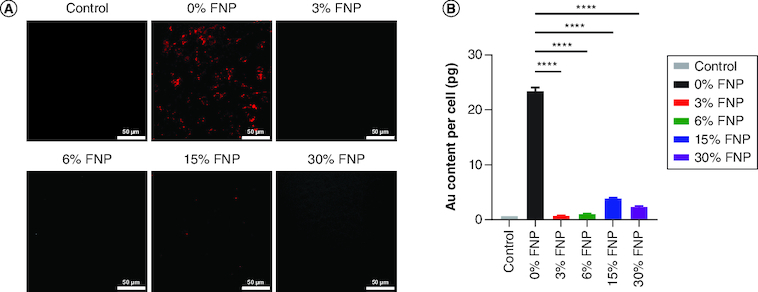
Cellular uptake of polymer-coated gold nanoparticles. **(A)** Dark field imaging of RAW 264.7 cells incubated with polymer-coated nanoparticles in OptiMem; red represents the gold content in cells (scale bar: 50 μm). **(B)** Quantitative analysis of polymer-coated nanoparticle uptake on RAW 264.7 cells using inductively coupled plasma optical emission spectroscopy. ****p ≤ 0.0001; ordinary one-way analysis of variance, Dunnett's multiple comparisons test, n = 3 independent experiments. FNP: Fluoro–gold nanoparticle.

To assess how fluorine affected phagocytosis, the authors incubated the five NPs (0, 3, 6, 15 and 30%, respectively), at a concentration of 50 μg/ml with mouse macrophage cells (RAW 264.7). Following a 24-h incubation period, they used dark field microscopy and ICP analysis of the cell lysates to examine phagocytic uptake (Figure 5). As shown in Figure 5A, the 0% FNPs displayed abundant phagocytic uptake when incubated with RAW 264.7 cells. Conversely, for 3, 6 and 30% FNPs, no visible uptake was detected. Surprisingly, a slight observable presence of phagocytic uptake was seen in the 15% FNP group. These overall observations were further validated by quantitative measurement of the gold content present inside RAW 264.7 cells using ICP-OES (Figure 5B). NP uptake in RAW 264.7 cells was greatest for the 0% FNPs (23.05 pg), while for the 3, 6 and 30% FNPs the presence of Au was more than tenfold less. Interestingly, here, too, the 15% fluorine NPs showed the highest uptake (3.84 pg) relative to the 0% fluorine NPs, potentially warranting further future investigation (Supplementary Table 4).

### Protein corona characterizations

**Figure 6.**
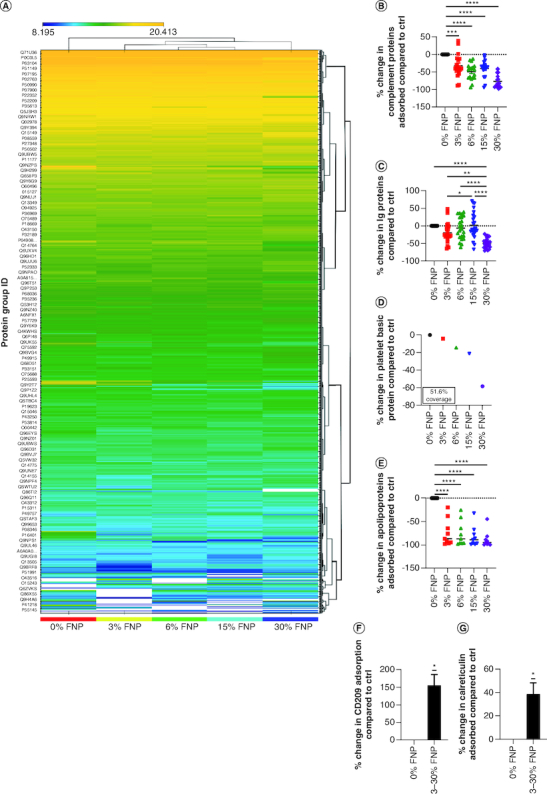
Protein adsorption determined by proteomic analysis. **(A)** Heat map for protein absorption. **(B)** Complement protein absorption compared with 0% fluorine. ***p ≤ 0.001; ****p ≤ 0.0001; ordinary one-way analysis of variance (ANOVA), Dunnett's multiple comparisons test. **(C)** Ig protein absorption compared with 0% fluorine. *p ≤ 0.05; **p ≤ 0.01; ****p ≤ 0.0001; ordinary one-way ANOVA, Tukey's multiple comparisons test. **(D)** Platelet basic protein absorption compared with 0% fluorine. **(E)** Apolipoprotein absorption compared with 0% fluorine. ****p ≤ 0.0001; ordinary one-way ANOVA, Dunnett's multiple comparisons test. **(F & G)** CD209 and calreticulin absorption compared with 0% fluorine, respectively.*p ≤ 0.05; one sample *t*-test. Ctrl: Control; FNP: Fluoro–gold nanoparticle.

Important studies have described how PEGylation significantly reduces protein adsorption of AuNPs; however, little has been reported on whether the addition of fluorine during polymer synthesis further reduces this effect [54], moreover if the percentage of fluorine added significantly impacts the adsorption of immunogenic proteins. To answer these questions, the authors adopted a proteomic approach to try to capture the interfacial interactions occurring between fluorine polymer-capped AuNPs and prominent immune-activating proteins prevailing in the blood. A complete picture of the proteins engaged in corona formation is illustrated in a heat map (Figure 6A).

Examination of the proteomic data revealed an overall decrease in protein adsorption of complement proteins in a percentage-dependent manor (p ≤ 0.001; p ≤ 0.0001), demonstrated as a negative log_2_ fold-change (Figure 6B). For the 3% fluorine NP group, an ∼30% reduction was observed, and for 6% fluorine group, a ∼50% reduction was observed. For the 15% and 30% fluorine groups, 40% and 79% reductions were demonstrated. On average, the reduction in protein affinity between the 3% and 30% was more than double (-33% and -77%, respectively), relative to the 0% fluorine polymer-capped NP control group. Complement C1q protein subunits (C1qB and C1qC) were reduced up to ∼85% in the presence of fluorine, with as little as 6% fluorine reducing adsorption by ∼43% (Supplementary Table 5). Additionally, the C1s protein was reduced by up to 63% for AuNPs coated with 30% fluorine polymers. An ∼30% for all other fluorine percentages was further demonstrated. The authors further identified a decrease in clusterin adsorption (protein coverage 44.3%; Supplementary Table 5) that was not related to fluorine percentage (-87, -87, -89, -92%, respectively).

The authors next examined the adsorption of natural antibodies, immunoglobulins IgM, IgG and IgA by comparing the adsorption of the different identified immunoglobulin fragments (n = 23 per group) notable variation was observed in the 3–15% (Figure 6C). This variation was particularly exaggerated in the 6% group. However, statistically, an increase in fluorine percentage between 3% and 15% and between 3% and 30% was demonstrated (p ≤ 0.05; p ≤ 0.01). The 30% fluorine group was most changed to the 0% control (∼50% less adsorption; p ≤ 0.0001). Meanwhile, platelet basic protein (51.6% coverage) was shown to reduce adsorption greater than twofold in relation to fluorine percentage (-4.15, -14.46, -21.31 and -58.55, respectively), relative to 0% fluorine controls (Figure 6D). Lipoprotein expression was also shown to be markedly reduced compared with the 0% fluorine control (Supplementary Table 6). Apolipoprotein E showed the highest overall protein coverage (76.3%) in the 0% fluorine control; however, the addition of fluorine produced ≥97% less adsorption, with only incremental (∼1%) decreases occurring between each group. Apolipoprotein A-I was also highly abundant in the 0% fluorine control (56.2%). However, unlike apolipoprotein E, apolipoprotein A-I adsorption was reduced in relation to fluorine percentage (-38, -64, -78, -89, respectively). Interestingly, in all instances, the reduction in adsorption was significant (p ≤ 0.0001) and in most subgroups increased in a relatively uniform manner regardless of the fluorine percentage.

Anti-inflammatory protein CD209 expression was significantly (p ≤ 0.05) increased in all of the fluorinated polymer groups (Figure 6F). Even with a 3% fluorine addition, protein adsorption was increased by 67%. In the 6–30% groups, >150% increase in CD209 expression was observed (Supplementary Table 7). Meanwhile, prophagocytic signaling protein calreticulin showed protein coverage of 50.6% and demonstrated a significantly increased adsorption in the fluorinated NPs compared with the 0% fluorine control (p ≤ 0.05; Figure 6G). While a modest 13% increase was observed for the 3% fluorine NPs, adsorption was about fourfold greater with the 6–30% fluorine NP groups (Supplementary Table 8).

## Discussion

In this study, the authors examined whether fluorine, as an addition to polymer-capped AuNPs, affected protein corona formation in relation to complement and other immune-reactive proteins residing in the blood. They first observed that although the molecules of the polymers had different sizes, this did not significantly affect the conversion rate. Therefore, affect the conversion rate thereby affirming that the method with varying feeding ratios was highly effective at producing polymers with a defined fluorine percentage, indicative of well-controlled RAFT polymerization. The authors further demonstrated the robust synthesis of FNPs with excellent stability. UV-VIS analysis and TEM results, visibly displaying a highly monodispersed population for each NP formula when immersed in H_2_O, demonstrate that both the size and shape were not significantly altered by either the addition of the polymer coat or the increase in percentage of fluorination.

NaCl is an intrinsic electrolyte making up 0.8% of the blood, where it helps maintain a healthy pH [55]. However, the presence of NaCl in a solution, depending on NP design, can readily promote aggregation if NPs are poorly stable, as well as influence protein corona formation and the likelihood of complement C3 protein adsorption [56–58]. Collectively, these factors can have a significant impact on the delivery and efficacy of the therapeutic cargo. For instance, Yang-hsin *et al.* for titanium dioxide and more recently Givens *et al.* for silicon dioxide NPs demonstrated significant aggregation in the presence of salts [57,59], Yang-hsin *et al.*, in particular, highlighting the potential to cause toxicity. Here the authors show that in PBS (representative of biological solution with 0.9% NaCl) the hydrodynamic size was significantly reduced in the FNPs compared with the nonfluorinated AuNPs, suggesting that fluorine helped prevent aggregation in biological buffer solutions. The authors attribute these results to the established hydrophobicity of fluorine and associated halogen bonds creating excellent colloidal stability, as also recently demonstrated by Wang *et al.* [60].

The complement system is a large group of well-established soluble and membrane-bound plasma proteins housed within the innate immune system. Much has been reported on the species and interspecies differences in complement activation and opsonization; therefore, protein analyses in the present experiments were performed using human serum to ensure complete relevance [61]. The complement system presents as the great nemesis for NP-led therapeutics [62]. Once activated, NPs may be phagocytosed through opsonization by complement protein (C3), the most abundant complement protein in the blood, as well as complement pattern-recognition molecules C1q and mannose-binding lectin [63–65]. Additionally, C3, C4 and C5 generate anaphylatoxins (C3a, C4a and C5a) and signal basophils to release large quantities of histamine capable of eliciting a systemic hypersensitivity reaction in patients, which can be lethal. In these investigations, fluorine was found to significantly reduce the adsorption of complement proteins in a percentage-dependent manor. This finding could be hugely important for NP-led treatments designed to fall under the immune system radar.

While complement protein synthesis is routinely attributed to the liver, studies have identified the presence of C1q in the brain, where it has been suggested that it adopts a neuroprotective role [66]. Therefore, NPs developed in the treatment of brain pathologies should take into account that excessive adsorption could overstimulate the classical complement pathway and ultimately prove detrimental to healthy brain cells. In this study, the authors identified that fluorine reduced the adsorption of C1q, suggesting that it may be able to play an immune-evasive role in NPs aimed at the brain. One potential caveat to these findings for complement proteins was for clusterin (protein coverage 44.3%), where a previous study by Aoyama *et al.* demonstrated that clusterin opsonization is highly important in imparting a stealth-like property to NPs – actively protecting against phagocytosis [67]. In the present study, the authors observed a decrease in adsorption of clusterin (Supplementary Table 4) regardless of fluorine percentage. However, when they exposed RAW 264.7 macrophage cells to the FNPs, fluorine was demonstrated to significantly prevent phagocytosis compared with the nonfluorinated control, suggesting that fluorine imparts an alternative mechanism of preventing phagocytosis. Taken together, these studies suggest that fluorine addition may help reduce complement protein adsorption, and thus cascade activation. Thus, the addition of fluorine may present as a simple way to modulate acute infusion reactions in polymer and lipid NP formulations and, as previously determined, may be a realistic design consideration for nanomedicines directed at brain diseases [68].

Natural antibodies IgM, IgG and IgA are commonplace in the blood and are said to increase with age [69]. Each is known to activate the complement system through binding to foreign materials [70,71], whereas platelet basic protein is a powerful chemoattractant and stimulus for neutrophils [72]. As essential first-line innate immune defense cells, neutrophils play an invaluable protective role against invading pathogens. However, as potent initiators of inflammation neutrophils, they can also impart damaging effects to healthy tissues [73]. Although potentially the least well-studied immune cells in relation to nanotoxicology, neutrophils have been observed to preferentially phagocytose PEG-based NPs in the blood [74]. Moreover, these cells are known to carry out a respiratory burst following stimulation, most notably by complement C5a, and encourage adaptive immune cells to mobilize [75]. In this study, the findings suggest an important role for fluorine in the preparation of PEGylated NPs to mitigate the negative effects of both immunoglobulin antibodies and neutrophils.

Apolipoproteins are routinely enriched in the protein coronas of engineered NPs and have been shown to highly influence NP effect *in vivo* [76,77]. Specifically, apolipoprotein-enriched coronas have been linked to cellular uptake and effective crossing of the blood–brain barrier; however, issues in relation to immunoreactivity remain a notable concern for NP studies that have adopted this approach [76,77]. This may be especially true for NPs that bind to apolipoprotein E, which are rapidly trafficked to the liver [33]. The present findings suggest that fluorine may work to slow down this process and thus help improve circulatory and target tissue times while reducing liver toxicity.

Within the blood, CD209 is a receptor protein found on monocytes (predendritic cells and macrophages). As a receptor on dendritic cells, CD209 is essential in recognizing several life-threatening viruses (HIV, Ebola, dengue and cytomegalovirus) and other pathogens (e.g., *Leishmania*, *Candida albicans*, *Mycobacterium tuberculosis*, *Streptococcus pneumoniae* and *Aspergillus fumigatus*) and presenting them to T cells [78]. While several studies have eloquently described how HIV and COVID viruses have hijacked the normal engulfment by dendritic cells as a way of spreading infection, studies have also demonstrated that NPs binding to CD209 could act as a microbicide-inhibiting viral spread of these infections [35,37]. Additionally, tumor glycoproteins have been identified as binding partners for CD209. In one study, CD209 on immature dendritic cells was demonstrated to adhere to colorectal cancer cells through the recognition of LewisX and LewisY antigens. This was in contrast to healthy colorectal cells with low expression of LewisX and LewisY, respectively [38]. Collectively, these studies identify how NP-binding CD209 has the potential to improve therapeutic outcome in a range of diverse pathologies. The data from the present study showed that the addition of fluorine encourages much greater adsorption of CD209 and thus could provide an eloquent and facile strategy for targeting colorectal cancers.

Cell death occurring due to combined chemotherapy/radiation facilitates the release of damage-associated molecular patterns, including calreticulin, in a process known as immunogenic cell death. Immunogenic cell death subsequently kick-starts the previously cancer-controlled immune system, resulting in increased populations of tumor antigen presenting and cytotoxic T cells [39,40]. However, calreticulin expression at the tumor site is variable and, as such, often suboptimal in initiating a robust antitumor immune response [79]. NPs present as an excellent with of maximizing calreticulin expression at the tumor site, as exemplified recently by Sethuraman *et al.* [39]. Sethuraman *et al.* developed lipid NPs with clone DNA of calreticulin encased within, delivered via intratumoral injection. The NPs, when used in combination with focused ultrasound, improved local and systemic antitumor immunity. Given the impressive adsorption increase observed in the presence of fluorine in the current study, fluorine could further be used as a way of increasing calreticulin expression at the tumor site. In this example, fluorine polymer-capped NPs could be doped with calreticulin *ex vivo* and NPs subsequently delivered locally to the tumor site, potentially offering an alternative to the Sethuraman *et al.* study that negates the need for DNA incorporation and the calreticulin protein to be expressed by cells.

Finally, the degree of hydrophobicity has been reported to dictate immune response both *in vitro* and *in vivo* [80]. While previous studies have suggested that hydrophobic NPs are more likely to promote protein accumulation on the surface, the present study demonstrated that the inclusion of fluorine up to 30% did not follow this theory [81]. In fact, 30% fluorine (the highest examined percentage) encouraged either the least or the greatest adsorption relative to the 0% fluorine polymer control in the proteins examined. Collectively, the remarkable decrease in phagocytosis by macrophages in combination with an overall reduction in complement protein interactions suggest that a superior ‘stealth-like’ behavior occurs as a consequence of the fluorine coating. However, it should be highlighted that the adsorption of some proteins is considered to yield better uptake, immune evasion and efficacy results *in vivo* [82]. In this respect, the present findings that clusterin is less adsorbed in the presence of fluorine require further investigations. One potential shortcoming of this the authors did not address the possible impact of blood flow on corona formation, which has been demonstrated to impact the composition and structure of protein coronas [83]. Experiments that systematically investigate the effects of flow will form the basis of the authors' ongoing research into how fluorine can be used to temper the effects of complement and other immune-reactive proteins.

## Conclusion

The protein corona effect remains one of the least well-understood phenomena affecting the intravenous delivery of nanomedicines. Currently, there is a lack of definitive information on how to manage the negative effects of the protein corona, specifically in relation to complement activation. Properly cataloguing how the building blocks used to synthesize polymer coatings interact with complement and additional immune-reactive proteins presents as one of the most effective ways of trying to address these challenges. In this study, we have taken important steps toward characterizing the immune protein corona fingerprint of fluorinated polymer-capped AuNPs and successfully illustrated a potential future role for fluorine in improving the intravenous delivery of gene-based theranostics.

## Supplementary Material

Supplementary Materials
