# Supplementary material for: Fluorine-modified polymers reduce the adsorption of immune-reactive proteins to PEGylated gold nanoparticles
Source: Nanomedicine (Lond). 2024 Apr 9;19(11):995–1012. doi: 10.2217/nnm-2023-0357 (PMC11221377; doi:10.2217/nnm-2023-0357)
Supplement: Supplementary Materials [file INNM_A_2339621_SM0001.zip › Supplementary tables.docx]

**Table S1:** Summary for polymer synthesis. Mn_BTPA-OH_ = 266.43, Mn_PFPE-BTPA_ = 2200, Mn_OEGA_ = 480, Mn_AIBN_ = 164.21

| **Polymers** | **CTA** | **M_CTA_ (mM)** | **m_CTA_ (mg)** | **M_OEGA_ (mM)** | **m_OEGA_ (mg)** | **M_AIBN_ (mM)** | **m_AIBN_ (mg)** |
| --- | --- | --- | --- | --- | --- | --- | --- |
| 0% F | BTPA | 0.751 | 200 | 30.027 | 14412.791 | 0.150 | 24.653 |
| 3% F | PFPE-BTPA | 0.091 | 200 | 7.909 | 3796.364 | 0.018 | 2.986 |
| 6% F | PFPE-BTPA | 0.091 | 200 | 3.636 | 1735.455 | 0.018 | 2.986 |
| 15% F | PFPE-BTPA | 0.227 | 500 | 3.409 | 1636.364 | 0.045 | 7.464 |
| 30% F | PFPE-BTPA | 0.227 | 500 | 1.136 | 545.455 | 0.045 | 7.464 |

**Table S2:** Polymer engraftment on gold nanoparticles by NMR.

| **Polymer coated AuNP** | **AuNP testing concentration (mg/mL)** | **Polymer (mg/mL)** | **F content (%)** |
| --- | --- | --- | --- |
| AuNP | N/A | N/A | N/A |
| 0% FNP | 0.2 | 0.17 | 0 |
| 3% FNP | 0.2 | 0.15 | 2.1 |
| 6% FNP | 0.2 | 0.10 | 3.3 |
| 15% FNP | 0.3 | 0.13 | 10.8 |
| 30% FNP | 0.3 | 0.20 | 20.0 |

**Table S3:** Interfacial intension of AuNP and FNPs.

| **Nanoparticle** | **Interfacial Tension at Water-Air interface (mN/m)** |
| --- | --- |
| AuNP | 64.91±1.15 |
| 0% FNP | 65.27±1.24 |
| 3% FNP | 54.36±0.16 |
| 6% FNP | 52.41±0.11 |
| 15% FNP | 53.89±0.33 |
| 30% FNP | 52.87±0.13 |

**Table S4:** List of complement proteins opsonised onto polymer capped gold nanoparticles and the % change with the addition of fluorine during polymer preparation.

| **Complement pathway proteins** | **Fluorine** | | | |
| --- | --- | --- | --- | --- |
|  | 3% | 6% | 15% | 30% |
| Complement C1q B | 31 | -43 | -65 | -90 |
| Complement C1q C | 39 | -40 | -36 | -83 |
| Complement C1s | -44 | -33 | -31 | -63 |
| Complement 2 | -55 | -43 | -36 | -51 |
| Complement C3 | 2 | -19 | -7 | -51 |
| Complement C4 | -36 | -37 | -29 | -71 |
| Complement C4 binding protein alpha chain | -30 | -33 | -36 | -85 |
| Complement C5 | -40 | -66 | -42 | -91 |
| Complement C7 | -43 | -67 | -34 | -93 |
| Complement C8 | -47 | -67 | -45 | -92 |
| Complement C8 alpha chain | -40 | -64 | -33 | -88 |
| Complement C9 | -12 | -58 | -21 | -90 |
| Vitamin K-dependent protein S | -16 | -16 | -17 | -42 |
| Complement component factor h-like 1 | -45 | -39 | -39 | -72 |
| Factor H | -60 | -70 | -64 | -93 |
| Clusterin | -87 | -87 | -89 | -92 |
| Complement factor B | -11 | -13 | -2 | -41 |
| Mannose-binding protein A | -89 | -94 | -94 | -98 |

**Table S5:** Quantitative analysis of FNPs uptake on RAW 264.7 cell lines using ICP-OES.

| **Nanoparticles treatment** | **Au content per cell (pg)** |
| --- | --- |
| N/A | 0.62 |
| 0% FNP | 23.05 ± 0.85 |
| 3% FNP | 0.66 ± 0.06 |
| 6% FNP | 1.00 ± 0.03 |
| 15% FNP | 3.84 ± 0.32 |
| 30% FNP | 2.28 ± 0.38 |

**Table S6:** List of apolipoproteins opsonised onto polymer capped AuNPs and the % change with the addition of fluorine during polymer preparation.

| **Lipoproteins** | **Fluorine** | | | |
| --- | --- | --- | --- | --- |
|  | 3% | 6% | 15% | 30% |
| Apolipoprotein A-I | -38 | -64 | -78 | -89 |
| Apolipoprotein A-IV | -93 | -96 | -97 | -99 |
| Apolipoprotein A-V | -95 | -96 | -97 | -97 |
| Apolipoprotein C-I | -76 | -78 | -86 | -95 |
| Apolipoprotein C-III | -65 | -41 | -68 | -82 |
| Apolipoprotein C-IV | -98 | -97 | -99 | -100 |
| Apolipoprotein E | -97 | -98 | -99 | -100 |
| Apolipoprotein L | -20 | -25 | -32 | -45 |

**Table S7:** List of receptor protein CD209 opsonised onto polymer capped AuNPs and the % change with the addition of fluorine during polymer preparation.

| **Receptor protein** | **Fluorine** | | | |
| --- | --- | --- | --- | --- |
|  | 3% | 6% | 15% | 30% |
| CD209 | 67 | 195 | 204 | 156 |

**Table S8:** Calreticulin opsonised onto polymer capped AuNPs and the % change with the addition of fluorine during polymer preparation.

| **Receptor protein** | **Fluorine** | | | |
| --- | --- | --- | --- | --- |
|  | 3% | 6% | 15% | 30% |
| Calreticulin | 13 | 47 | 38 | 58 |
